# The complex duration perception of emotional faces: effects of face direction

**DOI:** 10.3389/fpsyg.2015.00262

**Published:** 2015-03-20

**Authors:** Katrin M. Kliegl, Kerstin Limbrecht-Ecklundt, Lea Dürr, Harald C. Traue, Anke Huckauf

**Affiliations:** ^1^General Psychology, Institute of Psychology and Education, Ulm UniversityUlm, Germany; ^2^Medical Psychology, University Clinic of Psychosomatic Medicine and Psychotherapy, Ulm UniversityUlm, Germany

**Keywords:** time perception, duration estimation, emotional faces, perspective, sex congruence

## Abstract

The perceived duration of emotional face stimuli strongly depends on the expressed emotion. But, emotional faces also differ regarding a number of other features like gaze, face direction, or sex. Usually, these features have been controlled by only using pictures of female models with straight gaze and face direction. Doi and Shinohara (2009) reported that an overestimation of angry faces could only be found when the model’s gaze was oriented toward the observer. We aimed at replicating this effect for face direction. Moreover, we explored the effect of face direction on the duration perception sad faces. Controlling for the sex of the face model and the participant, female and male participants rated the duration of neutral, angry, and sad face stimuli of both sexes photographed from different perspectives in a bisection task. In line with current findings, we report a significant overestimation of angry compared to neutral face stimuli that was modulated by face direction. Moreover, the perceived duration of sad face stimuli did not differ from that of neutral faces and was not influenced by face direction. Furthermore, we found that faces of the opposite sex appeared to last longer than those of the same sex. This outcome is discussed with regards to stimulus parameters like the induced arousal, social relevance, and an evolutionary context.

## Introduction

There is growing evidence indicating that duration perception of emotional and neutral stimulus material is different (for reviews see, e.g., Droit-Volet and Meck, 2007; Droit-Volet and Gil, 2009; Schirmer, 2011). In detail, recent studies showed that emotional pictures (Angrilli et al., 1997; Grommet et al., 2011; Gil and Droit-Volet, 2012), emotional sounds (Noulhiane et al., 2007; Mella et al., 2011), and emotional videos (Loftus et al., 1987) are judged to last longer than respective neutral stimuli. Moreover, also the subjective duration of emotional faces differs from neutral ones (Droit-Volet et al., 2004; Effron et al., 2006; Tipples, 2008; Doi and Shinohara, 2009; Droit-Volet and Gil, 2009; Gil and Droit-Volet, 2011a,b). However, for faces there seems to be no general overestimation of emotional faces, but the effect strongly depends on the specific emotion being expressed. To this regard, Gil and Droit-Volet (2011a) report that facial expressions of anger, fear, sadness, and happiness go along with an overestimation of time, whereas the facial expression of disgust did not lead to any temporal distortion and the facial expression of shame even caused an underestimation of time (Gil and Droit-Volet, 2011c). Since the effectiveness of social interaction requires perpetual processing of temporal information (e.g., Droit-Volet and Meck, 2007; Chambon et al., 2008; Gil and Droit-Volet, 2011a), but also consists in interacting with people showing facial emotion expressions, it seems important to examine possible moderating variables.

In general, the most pronounced and stable effects consist in a duration overestimation of angry faces (Droit-Volet et al., 2004; Doi and Shinohara, 2009; Gil and Droit-Volet, 2011a,b). This is in line with the predictions of one of the most basic, but also most prominent time perception model: the pacemaker accumulator model (Treisman, 1963; Treisman and Brogan, 1992). In this model, arousal is thought to control the emission rate of temporal pulses sent by the pacemaker-like mechanism of an internal clock system. If arousal is high, then the pacemaker elicits more pulses and thus an interval is judged to last longer. Since studies showed that anger is a particularly arousing emotion (Russell and Mehrabian, 1977; Calder et al., 2001, 2004), it seems plausible that the pacemaker ticks faster, leading to longer duration perceptions when angry compared to neutral faces are presented (Droit-Volet et al., 2004; Droit-Volet and Gil, 2009; Gil and Droit-Volet, 2011a,b, 2012).

Yet, especially in real life situations, emotional faces do often not differ in regards of the presented emotion alone, but also regarding a number of other important features like gaze or face direction or sex. Although, in many of the studies examining duration perception of emotional faces, these features have been controlled by only using pictures of female models with straight gaze and face direction (e.g., Droit-Volet et al., 2004; Effron et al., 2006), Doi and Shinohara (2009) reported that the robust overestimation of angry faces could only be found when the model’s gaze was oriented toward the observer. They explain the gaze dependency of the duration overestimation for angry faces by differences in the induced level of arousal. Because gaze direction is usually considered to constitute an important cue for deducing the focus of attention of an interaction partner (Langton et al., 2000; Sander et al., 2007), an angry face gazing toward the observer might seem more relevant and might trigger higher fight-or-flight-reactions than an angry face with averted gaze (Sander et al., 2003). This is in line with many emotion theories suggesting that emotional stimuli possess high relevance for the survival and wellbeing of the observer (Brosch et al., 2010).

Given that differences in duration perception of emotional and neutral faces are indeed modulated by social relevance, the effect reported by Doi and Shinohara (2009) for gaze direction should also hold for face direction. In line with this, in an fMRI study, Sato et al. (2004) found higher activation in the amygdala, a brain region commonly associated to emotional processing (e.g., Breiter et al., 1996; Hariri et al., 2000, 2002; Whalen et al., 2001), when straight angry faces compared to averted angry faces were processed. Moreover, it has been argued that both, face as well as gaze direction are very important cues for social interaction (Argyle, 1990; Baron-Cohen et al., 1997). Thus, for instance, Baron-Cohen et al. (1997) found that the whole face is even more informative than either the eye or the mouth region alone, when recognizing basic emotions. Moreover, when asking participants to judge the gaze direction of a photographed face, Ricciardelli and Driver (2008) found different patterns of congruency effects depending on time constraints and on which parts of the face were visible. Based on this finding, they reasoned that the influence of the eye region might not be as dominant as previously assumed (Anstis et al., 1969). Using a stroop-task, Langton (2000) reported symmetrical interference effects of gaze and face direction and thus concluded that both cues consist of different systems of equal importance. Hence, it seems straightforward to replicate the study of Doi and Shinohara (2009), but examining the influence of face direction, instead of gaze direction, on duration perception of angry face stimuli.

In studies investigating the effect of emotional stimuli on perception, commonly a neutral condition and a small number of emotional conditions, often consisting of two basic emotions, are applied (e.g., Blair et al., 1999; Adams and Kleck, 2003, 2005; Effron et al., 2006; Doi and Shinohara, 2009; Tipples, 2011). In line with this, Doi and Shinohara (2009) did not only present neutral and angry faces, but also happy faces. Yet, there was no significant influence of gaze direction to happy faces. Assuming that the effect might be moderated by the amygdala as argued above (Sato et al., 2004), this seems reasonable, because the amygdala is often associated to the processing of negative stimuli (e.g., Blair et al., 1999; Hariri et al., 2000, 2002; Whalen et al., 2001). Thus, in the present study, we will use face stimuli showing neutral and two negative facial expressions, i.e., anger and sadness.

In order to draft a conceived hypothesis regarding the effect of changes in face direction to the duration perception of sad faces, a short excursion to research on emotional face processing is necessary: it has been argued that facial expressions of emotions should be characterized as examples of goal derived categories serving the goal of emotion communication (Barsalou, 1985; Horstmann, 2002; Brosch et al., 2010). Based on this, two distinct emotion categories can be distinguished: approach- and avoidance-oriented emotions. This distinction has been applied in numerous studies (e.g., Kleinke, 1986; Fehr and Exline, 1987; Elliot, 1999; Grumet, 1999; Adams and Kleck, 2003, 2005; Adams et al., 2006; Nelson et al., 2013; Rigato et al., 2013). Thus, it has been shown that approach-oriented emotions, like joy or anger, are often expressed with direct gaze, whereas avoidance-oriented emotions, like disgust or sadness, are often expressed with averted gaze (Kleinke, 1986; Fehr and Exline, 1987; Grumet, 1999). In line with these findings, studies report that detection of emotional expression and intensity ratings of emotional face stimuli are better and higher when integrative expression patterns of face and gaze are congruent in respect to the communicated goals and needs (Adams and Kleck, 2003, 2005; Cristinzio et al., 2010; Rigato et al., 2013). In detail, in the framework of the shared signal hypothesis, Adams and Kleck (2003, 2005) stated that when the gaze direction matches the underlying behavioral intent (approach vs. avoidance) communicated by an emotional expression, the perception of the respective emotion will be enhanced. Specifically, they showed that straight gaze enhances the perceived intensity of approach-oriented emotions like anger, whereas averted gaze enhances the perceived intensity of avoidance-oriented emotions like sadness (Adams and Kleck, 2003). Thus, according to the shared signal hypothesis, the reduced duration overestimation for angry face stimuli with averted gaze reported by Doi and Shinohara (2009) could also be explained because of a lower perceived intensity of these stimuli. If this is the case and the hypothesis holds also true for a modification of face direction instead of gaze direction alone, the reverse outcome should be observed for avoidance-oriented emotions like sadness implying that the duration of sad faces would be overestimated the more averted the gaze is.

Coming back to our primary interest in potential variations of duration perception of face stimuli depending on additional stimulus features, we also brought in the sex of the face stimulus before. This assumption is sustained by recent research. Chambon et al. (2008) found an interaction between the observer’s sex and the face model’s sex. Young observers perceived pictures of elderly models shorter than pictures of young models, only if the models and the observers were of the same sex. This effect is explained by a higher motivation to embody pictures showing persons of the same sex than persons of the different sex. In this line of research, embodiment is understood as the degree to which an observer mimics and imitates an emotional facial expression associated with feelings of identifying or showing empathy with the respective face model (Effron et al., 2006; Droit-Volet and Gil, 2009; Droit-Volet et al., 2013). Since these processes are discussed to convey the overestimation of angry face stimuli (Effron et al., 2006) as well as sad face stimuli (Gil and Droit-Volet, 2011a), also in the present study a moderating effect of sex can be assumed. Thus, in the present study respective effects were not controlled by presenting pictures of female models to female participants as in previous studies (e.g., Droit-Volet et al., 2004; Effron et al., 2006), but by using face stimuli of males and females and testing them on male as well as female participants.

To sum up, the present study pursues the following aims: first, we aim at examining the effect of face direction on duration judgments of angry face stimuli and thus test if the effect reported by Doi and Shinohara (2009) for gaze direction is similar for face direction. Second, we explore the effect of face direction to sad face stimuli and thus test predictions derived from the shared signal hypothesis (Adams and Kleck, 2003, 2005). Third, we additionally controlled for influences of the sex of the face model and the participant.

## Materials and Methods

### Participants

The sample consisted of 50 participants. 25 women (mean age *M* = 22.92, *SD* = 5.69) and 25 men (mean age *M*= 22, *SD*= 2.5) were recruited from the population of undergraduate students of Ulm University. They had normal or corrected-to-normal vision and received partial course credit for their attendance. All participants were naïve with respect to the experimental hypothesis and gave informed consent to participate in the study, which was conducted in accordance with the institutional ethical provisions and the Declaration of Helsinki.

### Apparatus

The experiment was programmed on a Windows computer with MATLAB, Version R2009b (The MathWorks) using the software library Psychtoolbox, Version 3.0.8 (Brainard, 1997; Pelli, 1997). Stimuli were presented on a 20^′′^ Vision Master Pro 512 monitor (1152 × 864 pixels) running at 100 Hz. A head-chin rest ensured a constant viewing distance of ∼60 cm at which the display subtended 36.87° by 28.07°. Left and right arrow keys of a standard keyboard served as response device.

### Stimuli

**FIGURE 1:**
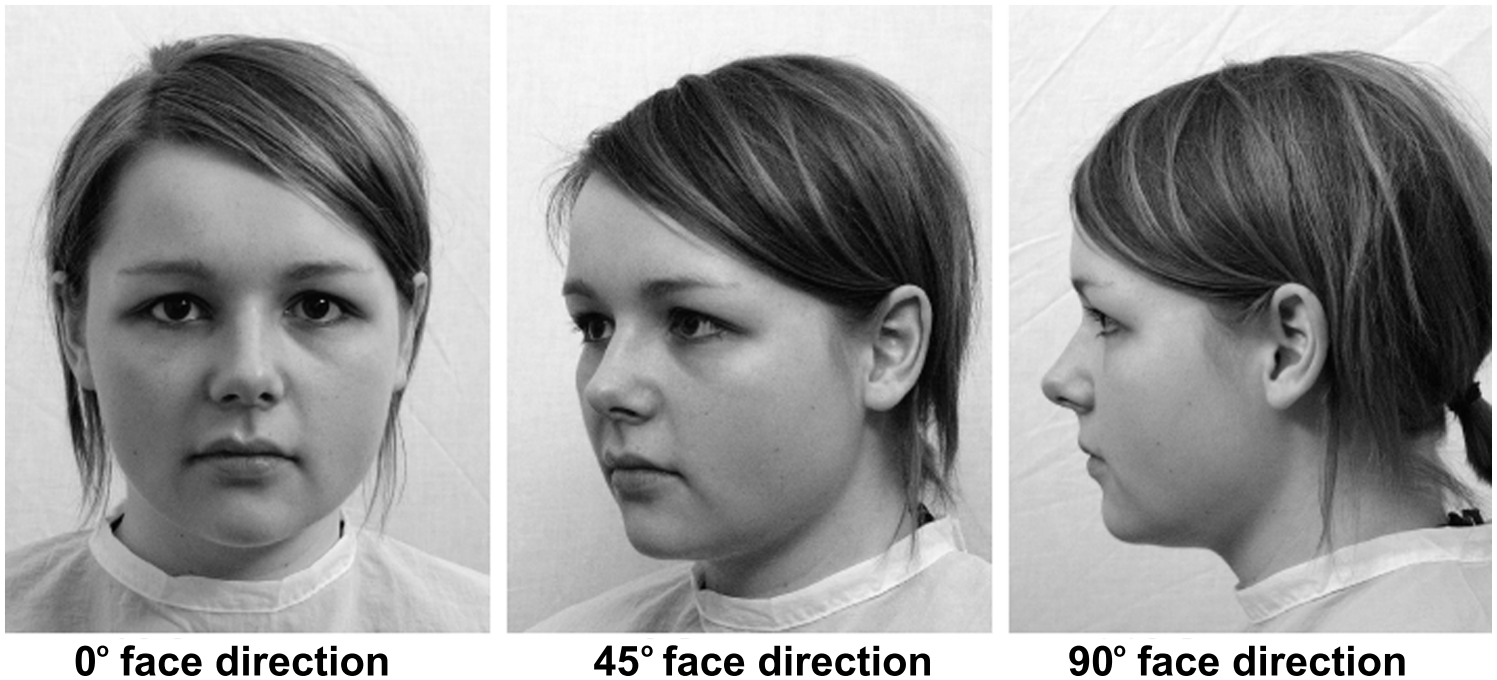
Illustration of a face stimulus showing model wSt36 with a neutral expression photographed with 0, 45, and 90^°^ face direction. Note that for the purpose of economical printing, the original stimuli were desaturated, whereas in the experiment, the stimuli were presented in the RGB color space.

Stimulus material was taken from the Pictures of Facial Affect – Ulm (PFA-U; Limbrecht et al., 2012; Limbrecht-Ecklundt et al., 2013). It consisted of neutral, angry, and sad emotional face expression stimuli of two male (mSt55, mSt57) and two female face models (wSt36, wSch49) photographed with face directions of 0, 45, and 90° aversion. As depicted in **Figure 1**, 0° face direction refers to straight view on the stimulus, 90° to profile view and 45° to the mean view between both. The stimuli had a mean luminance of 13.75 lx and covered an area of 6.77° × 9° in the center of a uniformly gray screen (13.4 lx, measured by a GOSSEN MAVOLUX 5032B USB luminance meter).

### Procedure

In general, the procedure consisted in a temporal bisection task, a commonly used task in duration studies (e.g., Droit-Volet et al., 2004; Tipples, 2008; Grommet et al., 2011; Kliegl et al., 2014). At first, participants were instructed verbally and in written form. Then, they were familiarized with the “short” (400 ms) and “long” (1600 ms) anchor durations by presenting dark gray rectangles (12.8 lx, 6.77° × 9°) in the screen center for the respective durations. In the following practice phase of 20 trials, each anchor stimulus was presented 10 times in randomized order and participants were asked to categorize these by pressing “K” (“kurz”: German for short) or “L” (“lang”: German for long) on a customary keyboard. Wrong answers were followed by a high-pitched beep. Practice trials were repeated, if a participant gave less than 90% correct answers.

**FIGURE 2:**
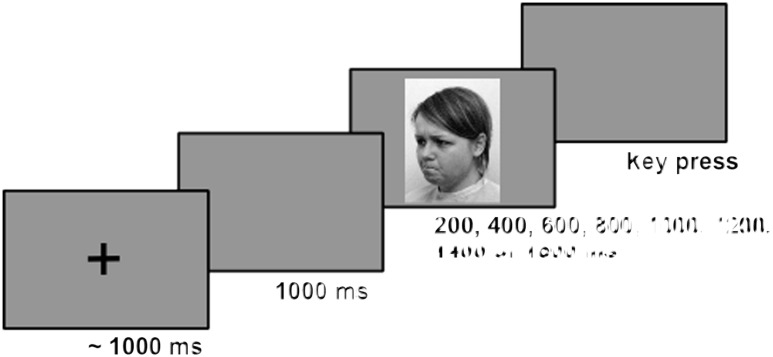
Illustration of a trial sequence. Here, the duration of a female face stimulus with an angry emotional expression photographed with a face direction of 45^°^ has to be rated.

During the following main experimental phase, no feedback was given and the rectangle was replaced by the face stimuli. Stimulus length varied between the anchor durations in steps of 200 ms resulting in stimulus durations of 400, 600, 800, 100, 1200, 1400 and 1600 ms. Participants were instructed to indicate whether the respective duration was closer to either the short or the long anchor which had been learned before. As illustrated in **Figure 2**, each trial started with a black fixation cross (1° × 1°, linewidth = 0.1°) presented in the center of an otherwise gray screen. In order to discourage rhythmical answering strategies (Povel and Essens, 1985), its duration was randomly drawn from a normal distribution with *M*= 1000 ms and *SD*= 250 ms, within fixed limits (min = 500 ms, max = 1500 ms). After a blank interval of 1000 ms, an emotional face stimulus was presented followed by a blank screen that remained visible until a new trial was started by the participant’s rating.

The experiment was run in two sessions of about 1 h each. The presentation order of the face stimuli was randomized within a session. The sex of the face model was nearly balanced with 13 women judging female faces, 12 women judging male faces, 12 men judging female faces and 13 men judging male faces. Each face stimulus was presented 10 times, resulting in 1260 trials for the 126 face expression – duration combinations (two models, three emotions, three face directions, seven durations). Thus, a session consisted of 630 trials. To prevent artifacts because of eye strain or fatigue, each testing session was split in nine blocks of 70 trials with breaks of about 1 min between the blocks.

### Results

**FIGURE 3:**
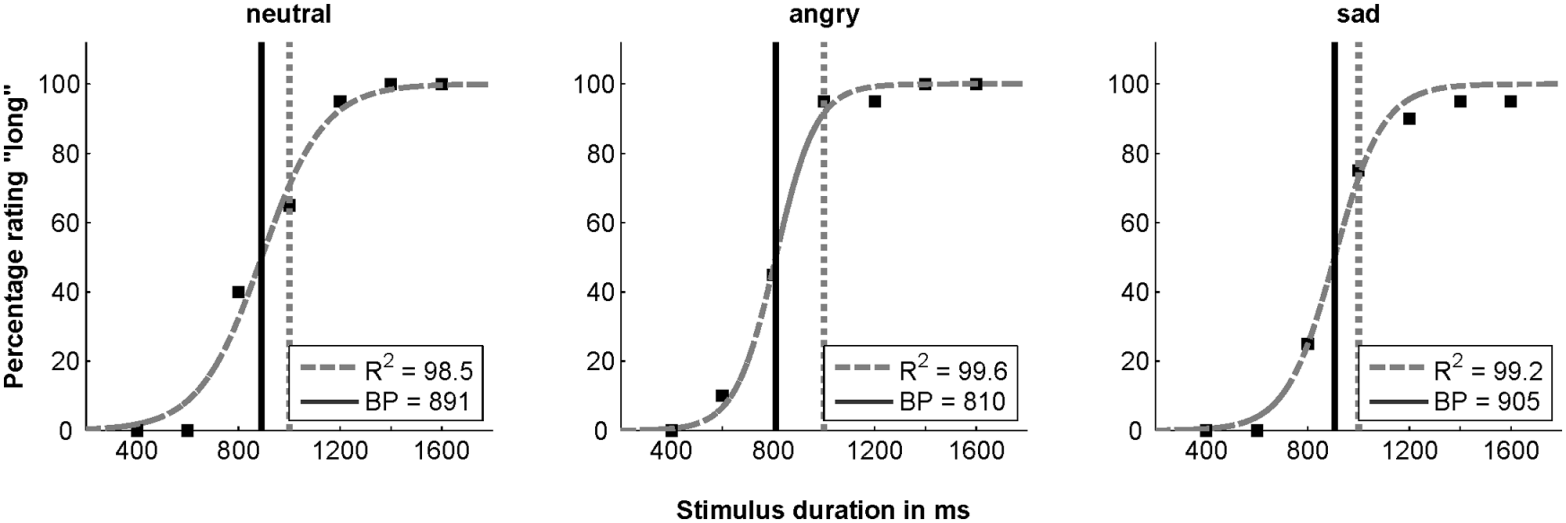
Ratings of a typical subject. Percentage of the “longer” ratings depending on the duration of the stimulus for neutral, angry and sad face stimuli photographed with 0^°^ face direction. Gray dashed curves show the fit. The vertical gray dotted lines indicate the objective BP of 1000 ms and the vertical black lines signal respective subjective BPs.

Data analysis was performed using MATLAB, Version R2009b (MathWorks, Inc.) and IBM SPSS Statistics 21 (IBM, SPSS Inc.). When the sphericity assumption was violated (*p* < 0.1), a Greenhouse-Geisser correction was performed. For each participant, the bisection point (BP) of each experimental condition (emotion × face direction) was determined. The BP is a commonly used measure in time perception (Grondin, 2008). It is defined as the 50% threshold at which the subject shows a maximum of uncertainty when estimating the duration of a stimulus. It is calculated by fitting a logistic function to the observed relation between the “long” ratings and the actual stimulus durations (compare Tipples, 2008; Kliegl and Huckauf, 2014). This procedure is graphically illustrated in **Figure 3** for stimuli presented with 0° face direction. If the objective and the subjective duration coincide, the gray and the black vertical lines lie on top of each other at 1000 ms. A shift to the left on the *x*-axis indicates an overestimation of the stimulus, as in the example, an angry face has to be presented for 810 ms in order to equal the objective threshold.

To confirm the reliability of the fitting procedure and thus the BPs, the goodness of fit was analyzed by calculating *R*^2^ values. As summarized in **Table 1**, the fits were sufficiently good and did not show any trends depending on the condition.

**Table 1:** Mean *R^2^* describing the goodness of fit.

Emotion	Neutral	Angry	Sad
0^°^ face direction	0.98 (0.01), range: 0.94–1.00	0.99 (0.02), range: 0.88–1.00	0.99 (0.01), range: 0.95–1.00
45^°^ face direction	0.98 (0.02), range: 0.89–1.00	0.99 (0.02), range: 0.89–1.00	0.99 (0.02), range: 0.89–1.00
90^°^ face direction	0.99 (0.01), range: 0.94–1.00	0.98 (0.01), range: 0.93–1.00	0.98 (0.01), range: 0.93–1.00

*For each subject fitting was conducted separately in each of the nine experimental conditions. Values in brackets indicate the respective standard deviations (*SD*s). The range names the minimum and maximum value in the respective condition*.

**FIGURE 4:**
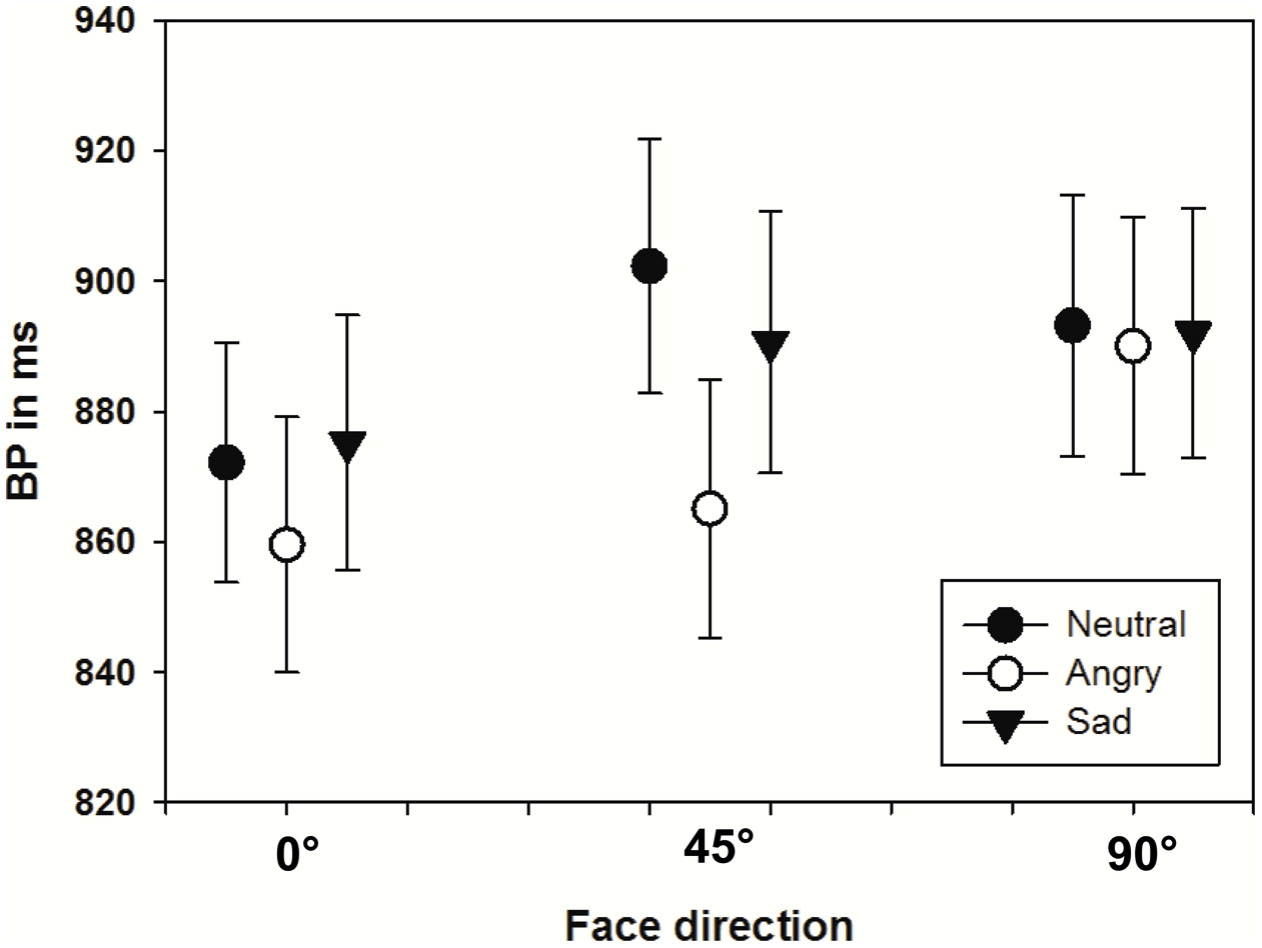
Mean BPs depending on the presented emotional expression (neutral, sadness or anger) as well as the face direction (0, 45, or 90^°^ aversion). Error bars indicate the standard error (*SE*).

Bisection point values were analyzed using a repeated measures ANOVA with the within subject factors emotion (neutral, angry, sad) and face direction (0, 45, 90°) as well as the between subject factors sex of the face model and sex of the observer. Statistical results showed a significant main effect of emotion [*F*(2,92) = 9.65, *p* < 0.001, $\eta_{p}^{2}$ = .17] with *post hoc* contrasts using the neutral category as reference indicating that angry faces were overestimated [*F*(1,46) = 15.63, *p* < 0.001, $\eta_{p}^{2}$ = 0.25], whereas sad faces were not [*F*(1,46) = 0.72, *p* > 0.05]. Moreover, the analysis revealed a significant main effect of face direction [*F*(2, 92) = 14.19, *p* < 0.001, $\eta_{p}^{2}$ = 0.24] with tests of sequential within-subjects contrasts showing significant differences between consecutive face directions [0–45°: *F*(1,46) = 13.32, *p* < 0.001, $\eta_{p}^{2}$ = 0.23; 45–90°: *F*(1,46) = 15.26, *p* < 0.001, $\eta_{p}^{2}$ = 0.25]. However, when interpreting these results also the significant interaction between the factors perspective and emotion has to be considered [*F*(2,184) = 3.11, *p* < 0.05, $\eta_{p}^{2}$ = 0.06]. As illustrated in **Figure 4**, this interaction with a comparatively small effect size derives mainly from the maximally diverse duration ratings of neutral, sad, and angry faces in the 45° condition, but does not reverse the reported main effects in general.

**FIGURE 5:**
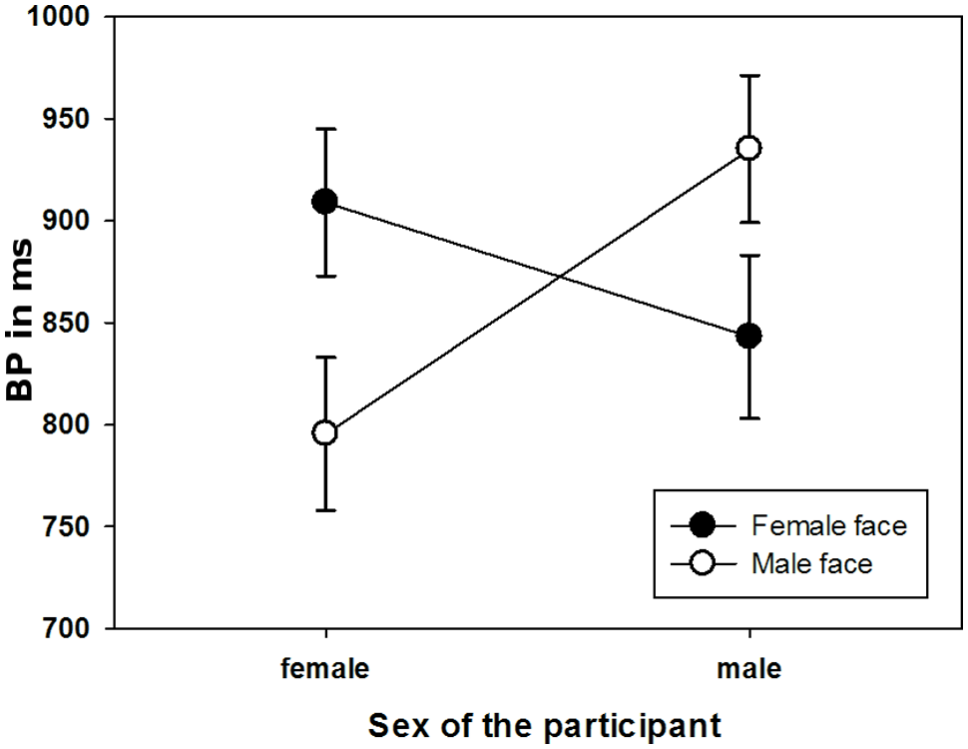
Mean BPs illustrating the significant interaction between sex of the participant and the sex of the face model. Error bars indicate the standard error (*SE*).

Furthermore, the interaction between the sex of the face model and sex of the participant was significant [*F*(1,46) = 5.66, *p* < 0.05, $\eta_{p}^{2}$ = 0.11). As depicted in **Figure 5**, women judged male faces to last longer than female ones and vice versa for men. All further main effects and interactions did not reach significance.

In order to examine the influence of face direction on the duration perception of angry and sad faces more closely, we subsequently computed two separate ANOVAs for the respective emotion conditions with the within subject factor face direction. To counteract the problem of multiple comparisons, Bonferoni corrections were used resulting in an α of 0.025. This analysis revealed a significant effect of face direction on the duration estimation of angry faces [*F*(2,98) = 10.12, *p* < 0.025, $\eta_{p}^{2}$ = 0.12], but no effect on the duration estimation of sad faces [*F*(2,98) = 3.19, *p* > 0.025]. As illustrated in **Figure 4**, with increasing face aversion, there is a decrease in the duration overestimation for angry faces and a similar, but less pronounced, trend for sad faces.

## Discussion

The goal of this experiment was threefold: first, we examined the effect of face direction on duration judgments of angry faces testing if the effect reported by Doi and Shinohara (2009) for gaze direction generalizes to face direction. Second, we explored the effect of face direction to sad face stimuli testing predictions derived from the shared signal hypothesis (Adams and Kleck, 2003, 2005). Third, we additionally controlled for influences of the sex of the face model and the participant.

The present experiment replicated the temporal overestimation of angry faces compared to neutral faces that has been reported in a number of recent studies (e.g., Droit-Volet et al., 2004; Tipples, 2008, 2011; Gil and Droit-Volet, 2011a,b). With respect to our first goal, we showed that the temporal overestimation of angry facial expressions is also influenced by face direction as it is by gaze direction (Doi and Shinohara, 2009). Thus, the duration overestimation was maximal if the angry face was directed to the observer and declined the more averted it was. As has been argued in the introduction, this outcome seems reasonable since both, eye as well as gaze direction, constitute important cues for social interaction (Argyle, 1990; Baron-Cohen et al., 1997). Looking away as well as turning away usually signals withdrawal of social attention. Therefore, the emotional state of an averted interaction partner can be assumed to be of less social relevance (Sander et al., 2003), because his emotional expression might not be directed to the observer, but to another person. Consequently, when looking at a straight angry face, the prompt initiation of fight-or-flight-reactions is more important compared to when looking at an averted angry face. Furthermore, the turning of the whole body, including the eyes, constitutes an even stronger and more consistent social cue than mere gaze shifts (Langton, 2000; Aviezer et al., 2012). Thus, when Langton (2000) placed social information from gaze and head orientation into conflict, he found symmetrical interference indicating that both cues mutually influence the analysis of the dialog partner’s social attention.

Exceeding the work of Doi and Shinohara (2009), we presented three graduations of aversion instead of two. Because we observed a stepwise increment of the duration overestimation over the three conditions with maximal values for straight face direction and minimal ratings for 90° face direction, we can figure the shape of the effect more precisely. Furthermore, the stimulus material used by Doi and Shinohara (2009) was graphically adapted by replacing the model’s irises in order to create emotional face stimuli with straight and averted gaze. As discussed by the authors, especially the averted gaze stimuli might have appeared a little unnatural. Especially, since it has been argued that the eye region is particularly important when processing emotional face stimuli (e.g., Anstis et al., 1969), this limitation could have influenced the results, because rare stimuli are perceived differently (Tse et al., 2004; Pariyadath and Eagleman, 2007). Thus, with our study we were able to underline the reliability of the effect and to generalize it to a slightly different stimulus material, since we observed the same pattern of results for stimuli consisting in original photographs taken simultaneously from different face direction (Limbrecht-Ecklundt et al., 2013).

With the direction dependency now observed in two studies using slightly different stimuli, it might be concluded that not the perception of the face stimulus *per se*, but its interpretation in the social context, might modulate specific arousal states and thus might lead to modulations in duration perception.

Regarding our second aim, the duration estimates of sad face stimuli did not differ from duration estimates of neutral face stimuli and no modulation by face direction was found. However, the reported overestimation in previous papers (Droit-Volet et al., 2004; Gil and Droit-Volet, 2011a) was not very obvious and in a recent paper Droit-Volet et al. (2013) even state that the effect of sadness is not clear.

With respect to the shared signal hypothesis (Adams and Kleck, 2003, 2005; Adams et al., 2006), this outcome questions the transferability of respective predictions to the domain of duration perception: on the one hand, the observed face direction dependence of the duration estimation of angry facial expressions fits into this framework, because the perceptual intensity and thus also duration perception of facial expressions showing an approach-oriented emotion like anger can be assumed to decrease with increasing aversion of the face (Adams and Kleck, 2003, 2005; Adams et al., 2006). However, on the other hand, the shared signal hypothesis anticipates higher perceptual intensity and thus also longer duration perception for averted facial expressions showing avoidance-oriented emotions like sadness. If this pattern held true, it would have allowed a substantial facilitation in predicting the modulations in duration estimations of emotional face stimuli by varying face directions. Yet, the assumed pattern of increasing duration perception with growing aversion of sad facial stimuli was not observed here. To the contrary, the results even showed a trend in the opposite direction, i.e., decreasing duration perception with growing aversion.

Thus, although the distinction between approach- and avoidance-oriented emotions has successfully been applied in many studies (e.g., Elliot, 1999; Adams et al., 2006; Nelson et al., 2013; Rigato et al., 2013) and has proven to be fundamental when differentiating the influence of gaze direction on recognition performance of emotional face stimuli (Adams and Kleck, 2003, 2005), this distinction does not seem helpful when trying to forecast duration estimations.

Yet, originally the shared signal hypothesis was developed with modulations of gaze instead of modulations of face direction. Thus, our conclusions have to be restricted to this stimulus parameter. Moreover, considering the feeble effect of sad face stimuli, one might argue that the choice of sad faces representing the avoidance category was not beneficial and that a different effect would emerge when using fearful face stimuli, instead. Yet, although fearful face stimuli probably cause more pronounced temporal overestimation (e.g., Droit-Volet et al., 2013), the predictions of the shared signal theory should hold true for any emotion comprised in this category.

In contrast to our hypothesis derived from the shared signal hypothesis (Adams and Kleck, 2003, 2005; Adams et al., 2006), we observed a main effect indicating that over all emotions the perceived durations decreased with increasing aversion of the face stimulus. This can be understood in the framework of social attention, too: as described above, turning to somebody is commonly interpret as a social prompt with stimulative character (Argyle, 1990; Sander et al., 2003) that might lead to an increased arousal level of the observer that in turn might accelerate the ticking rate of the “inner clock” (e.g., Treisman, 1963; Droit-Volet et al., 2004; Droit-Volet and Gil, 2009), whereas turning away might lead to the contrary.

Third, we aimed for controlling influences of the sex of the face model and the participant. In this context, the results revealed an interaction between the observer’s and the model’s sex. In general, stimuli showing a model of the opposite sex were perceived to last longer than stimuli showing a model of the same sex. Thus, men rated female face stimuli to last longer than male face stimuli and women rated male face stimuli to last longer than female face stimuli. A similar interaction effect moderated by the observer’s sex has already been reported by Chambon et al. (2008). As outlined in the Introduction, in this study, participants judged the duration of pictures showing a young person longer compared to pictures showing elderly persons only when they shared the same sex. This effect is explained by a higher identification with persons of the same sex and thus also a higher motivation to embody the perceived person (i.e., slow movement of elderly). Thereby embodiment is understood as the degree to which an observer mimics and imitates an emotional facial expression associated with feelings of identifying or showing empathy with the respective face model (Effron et al., 2006; Droit-Volet and Gil, 2009; Droit-Volet et al., 2013). Yet, this explication does not picture our results adequately, since it would forecast a three-way-interaction between the sex of the observer, the sex of the model and the depicted emotion: if sharing the same sex increased the embodiment, one should expect more pronounced effects of emotional stimuli in the respective conditions, which was not found here.

Instead, the observed overestimation of sex congruent photographs might again be explained by induced arousal, specifically in the evolutionary context of dating: especially young people in the reproductive age like the participants in our study are likely to check interaction partners for being possible mates (Buss, 2005). Ensuing from this viewpoint, photographs of the opposite sex might be more arousing and also socially more relevant. This in turn, could cause an acceleration of the “internal clock” leading to longer duration perception (Treisman, 1963; Treisman and Brogan, 1992; Droit-Volet et al., 2004; Droit-Volet and Gil, 2009; Gil and Droit-Volet, 2011b, 2012). Because, in the context of dating, arousal and social relevance might similarly be influenced by attractiveness, this explanation is substantiated by Ogden (2013)’s findings which show that participants overestimate the temporal duration of attractive faces in contrast to unattractive faces. However, since arousal or attractiveness ratings of the faces were not obtained, this explanation remains preliminary.

Regarding elicited arousal as the crucial moderator variable, an interaction between the sex of the observer and the depicted emotion could also be expected, since studies suggest that men and women process emotional stimuli differently (Kring and Gordon, 1998; Bradley et al., 2001; Canli et al., 2002; Hamann and Canli, 2004; Schirmer et al., 2006). More in detail, for example, Bradley et al. (2001) and Canli et al. (2002) found that men show lower arousal in response to aversive images than women. Yet, a respective interaction was not observed in the present study.

Furthermore, a significant main effect of the observer’s sex on duration estimations was not observed. This is in contrast to several studies that reported differences in duration estimations between men and woman (e.g., Espinosa-Fernández et al., 2003; Hancock and Rausch, 2010; Rammsayer and Troche, 2010). However, the general picture is not very clear and there is also a number of contrasting results (for a review see Block et al., 2000). This heterogeneity is often explained by differences in the used tasks and the length of the durations in focus (e.g., Hancock and Rausch, 2010). Thus, Rammsayer and Troche (2010) observed different performance levels for men and women only in rhythm perception and temporal discrimination tasks using empty intervals, but equal performance in temporal-order judgments, temporal generalization and temporal discrimination tasks using filled intervals. Moreover, using an interval reproduction task Espinosa-Fernández et al. (2003) found that men and women performed equally for shorter intervals (10 s), whereas women underproduced long intervals (1 and 5 min). Considering that we used a bisection task (closely related to a temporal generalization task; compare Grondin, 2010) with filled intervals of 1 s in average, our results are in line with previous research.

These results appear particularly interesting since many studies in this field only tested females (Droit-Volet et al., 2004; Effron et al., 2006), much more females than males (Tipples, 2008, 2011) or did not report the sex of the observers (Gil and Droit-Volet, 2011a,b). Moreover, also in the studies that included females and male participants (Tipples, 2008, 2011; Doi and Shinohara, 2009), effects of the observer’s sex were not analyzed probably due to the small number of tested male subjects. Furthermore, stimuli mainly consisted in photographs of female faces (Droit-Volet et al., 2004; Effron et al., 2006; Gil and Droit-Volet, 2011a,b). Thus, to our knowledge, this is the first study that systematically controls and analyzes the effect of the sex of the participant and the face model on duration perception of emotional face stimuli. However, one could argue that also in the present study each of the four experimental groups only comprised 12 and 13 participants, respectively. Yet, many experimental psychological studies are based on similar sample sizes (e.g., Doi and Shinohara, 2009; Gil and Droit-Volet, 2011b; Kliegl and Huckauf, 2014). For instance, Doi and Shinohara (2009) only tested 11 participants and observed a well explainable and approved effect.

## Conclusion

To sum up, in line with current findings, we report a significant overestimation of angry compared to neutral face stimuli that was modulated by face direction. This replicates results of Doi and Shinohara (2009) reported for gaze direction and suggests a generalization of the findings with respect to social relevance. Moreover, the perceived duration of sad face stimuli did not differ from that of neutral faces and was not influenced by face direction. Furthermore, we found that faces of the opposite sex appear to last longer than those of the same sex. These outcomes, taken together, draw a complex picture of the factors influencing duration perception. It seems crucial to take account of the meaning of an emotional stimulus in the social context, especially considering social relevance, when trying to understand and forecast its perceived duration.

From a theoretical view, changed duration perceptions due to social context and relevance might be attributed to changes in the ticking rate of our “inner clock” (Treisman, 1963). However, the designated moderator variable, i.e., the evoked arousal, has not been examined in the present study. Future studies should examine this influence for instance by verbal self-assessment as well as biophysiological markers.

## Author Contributions

KK: idea and leading conception of the work, programming of the experiment, data collection and in charge for the analysis as well as the interpretation of the results. KK is the leading writer of the manuscript and agrees with and is accountable for all aspects of the work in ensuring that questions related to the accuracy or integrity of any part of the work are appropriately investigated and resolved.

KL-E: idea and intellectual input to the experiment planning, particularly in respect of providing, pre-analysis, validity and selection of stimulus material and critically revising earlier versions of the manuscript. KL-E agrees with and is accountable for all aspects of the work in ensuring that questions related to the accuracy or integrity of any part of the work are appropriately investigated and resolved.

LD: substantial contributions to data collection and analysis as well as significant contributions to the writing of the manuscript. LD agrees with and is accountable for all aspects of the work in ensuring that questions related to the accuracy or integrity of any part of the work are appropriately investigated and resolved.

HT: intellectual input to the experiment planning, particularly in respect of providing, pre-analysis, validity and selection of stimulus material and critically revising the work. HT agrees with and is accountable for all aspects of the work in ensuring that questions related to the accuracy or integrity of any part of the work are appropriately investigated and resolved.

AH: intellectual input to the conception of the work and critically revising the analysis and interpretation of results as well as earlier versions of the manuscript. AH agrees with and is accountable for all aspects of the work in ensuring that questions related to the accuracy or integrity of any part of the work are appropriately investigated and resolved.
